# Lipopolysaccharide induced systemic inflammation and heart rate variability in a term newborn piglet model

**DOI:** 10.1038/s41390-024-03394-y

**Published:** 2024-07-28

**Authors:** Mette Vestergård Pedersen, Ann Frida Videbæk Renberg, Julie Kehlet Christensen, Hannah Brogaard Andersen, Ted Carl Kejlberg Andelius, Kasper Jacobsen Kyng, Mads Andersen, Tine Brink Henriksen

**Affiliations:** 1 Department of Clinical Medicine, Aarhus University, Aarhus, Denmark; 2 Department of Pediatrics, Aarhus University Hospital, Aarhus, Denmark

## Abstract

**Background:**

Early biomarkers are needed to improve diagnosis and support antibiotic stewardship in neonatal sepsis. Heart rate variability (HRV) is proposed as such a biomarker. However, there is a lack of studies in term newborns. Infusion of lipopolysaccharide (LPS) from *Escherichia coli* induces systemic inflammation comparable to sepsis in newborns. We aimed to study the effect of systemic LPS induced inflammation on HRV in term newborn piglets.

**Methods:**

Baseline HRV was recorded for 1 h. This control period was compared to the hourly HRV for each piglet (*n* = 9) during 4 h of LPS infusion. For comparison, we used a mixed-effects regression model.

**Results:**

Systemic inflammation induced by LPS was found to reduce HRV. Compared to baseline, most measures of HRV decreased to lower values compared to baseline at 2 h, 3 h, and 4 h after initiation of LPS infusion. Heart rate (HR) was increased at 2 h, 3 h, and 4 h. When adjusting for HR in the mixed-effects regression model all reductions in HRV were explained by the increase in HR.

**Conclusions:**

Reduced HRV may be an early biomarker of neonatal sepsis. However, an increase in HR alone could be an already available, more accessible, and interpretable biomarker of sepsis in term neonates.

**Impact:**

- In a term newborn piglet model, systemic inflammation induced by lipopolysaccharide from *Escherichia coli* reduced heart rate variability measures and increased heart rate.
- All reductions in heart rate variability were mediated by heart rate.
- While heart rate variability may be a biomarker of sepsis in term newborns, changes in heart rate alone could be a more readily available biomarker.

## Introduction

Sepsis is an acute critical condition caused by a dysregulated systemic inflammatory response to a bacterial infection. The World Health Organization estimates 15% of all neonatal deaths worldwide to be caused by neonatal sepsis.^1,2^ Sepsis in term newborns is often caused by vertical transmission of pathogens such as Group B *Streptococci* (GBS) or Gram-negative rods, such as *Escherichia coli* (E. coli).^3^ As the condition may progress rapidly towards multi-organ failure and shock, early diagnosis and treatment is essential in order to reduce morbidity and mortality.^4^

No specific clinical signs define neonatal sepsis, which makes accurate and timely diagnosis challenging.^5–7^ Early identification of neonatal sepsis is primarily based on clinical presentation which is nonspecific or sometimes asymptomatic.^8–10^ Blood culture is the golden standard of diagnosis, but limited by long waiting time and risk of false negative results.^11–14^ Due to the potential rapid development and severe course, empirical antibiotic therapy may be started based on vague clinical suspicions. Improvement of sepsis diagnostics by early biomarkers or diagnostic tools is important to improve timely diagnosis and to guide the use of antibiotics.^4,15^

Heart rate variability (HRV) has been proposed as an early biomarker of late onset neonatal sepsis.^16–18^ HRV describes the variation in time interval between successive heart beats; the so called RR-intervals.^19^ The variation is complex and constantly influenced by many factors, with the autonomic nervous system thought to be the main factor.^17,19,20^ HRV may be used to predict late onset sepsis and sepsis-like illness in neonates preceding diagnosis from clinical assessment alone.^18,21^ Previous findings are limited to cover late onset sepsis in preterm and VLBW neonates where HRV monitoring was associated with improved mortality.^18,22,23^ The potential use of HRV as a biomarker in term neonates remains understudied.

An experimental animal model allows for standardized conditions and reproducibility, which makes it possible to reduce bias and confounding from uncontrolled factors that may also affect HRV in human newborns. Lipopolysaccharide (LPS) is an endotoxin from the cell membrane of the bacteria E. coli. When administered intravenously it induces endotoxemia; a systemic inflammatory sepsis-like condition with hemodynamic and metabolic changes comparable to those found in patients with Gram negative sepsis.^24–27^ To mimic the potential effect of sepsis on HRV in term human newborns, we used a term newborn piglet model exposed to LPS. The aim of this study was to investigate the effect of systemic inflammation established by continuous infusion of LPS on HRV in a term newborn piglet model.

## Methods

### Ethical statement

The experiments were approved by the Danish Animal Experiments Inspectorate (2018-15-0201-01555) and conducted and reported in accordance with the ARRIVE 2.0 guidelines (ARRIVE checklist is presented in Supplementary material).^28^

### Experimental procedures

**Fig. 1:**
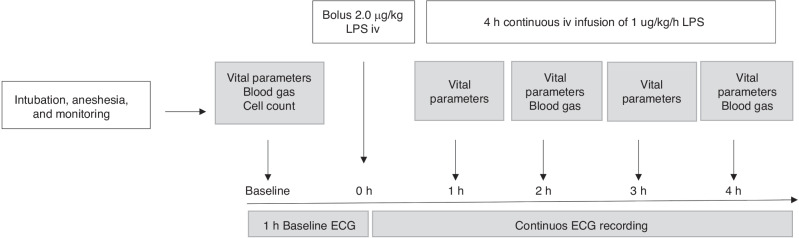
Study overview. LPS Lipopolysaccharide from *Escherichia Coli*. ECG electrocardiogram.

This study is a secondary analysis of ECG data from an experiment designed for other outcomes. ECG was recorded for 1 h of baseline, then LPS infusion was initiated, and ECG recorded the following 4 h during LPS infusion. Each animal served as its own control when comparing each hour during LPS infusion to baseline. A timeline of the experimental procedures is presented in Fig. 1.

The animals used in the experiments were Danish Landrace piglets, less than 12 h of age and of either sex. The animals were provided from herds included in a health-monitoring program, where animals are screened for different pathogens that could impact pigs in a productive setting. As per our protocol animals were excluded if they showed any signs of illness, weakness, or trauma prior to the experiment. The experimental procedures are described in detail elsewhere and are summarized in short.^29^

#### Anesthesia and monitoring

Anesthesia was initiated with sevoflurane inhalation 2–4%. Peripheral intravenous (iv) access was obtained through an ear vein. A bolus of propofol 10 mg/kg, fentanyl 30 μg/kg, and rocuronium 1 mg/kg was administered iv. The animals were intubated and mechanically ventilated with settings adjusted to reach and maintain a target end-tidal CO_2_ of 4.5–5.5 kPa. Throughout the experiments, anesthesia and analgesia was maintained with iv infusion of propofol 4–12 mg/kg/h and fentanyl 5–10 μg/kg/h. To maintain glucose and electrolytes, the animals received iv infusion of 10 ml/kg/h of NeoKNag (15 mmol/L Na + , 25 mmol/L Cl-, 10 mmol/L K + , 505 mmol/L glucose). Initial prophylactic antibiotic treatment of gentamycin 5 mg/kg and ampicillin 30 mg/kg was administered iv during baseline. Rectal temperature was measured continuously with a target normothermic temperature for piglets between 38.5–39 °C. Temperature was maintained by an air heated mattress.

#### Infusion of lipopolysaccharides

After 1 h of baseline recording, the animals received an iv bolus of 2.0 μg/kg LPS from *E. coli* (Serotype 0111:B4, Sigma-Aldrich, St. Louis, Mo.) to induce a sepsis-like condition. This was followed by 1.0 μg/kg/h continuous infusion for 4 h. These doses were used in previous piglet studies to create an inflammatory response comparable to sepsis.^30,31^

#### Blood samples and vital signs

A central arterial and venous access was secured by inserting umbilical catheters with aseptic technique. These were used for continuous monitoring of mean arterial blood pressure (MABP), blood sampling, and to administer drugs. Blood samples were transferred to EDTA glass tubes and analyzed for total white blood cell count, neutrophil count, platelet count, and hemoglobin concentration. The samples were analyzed at baseline and 4 h after initiation of LPS infusion using IDEXX ProCyte Dx Hematology Analyser (IDEXX Laboratories, Inc.). Arterial blood samples were obtained at baseline, 2 h and 4 h after initiation of LPS infusion to monitor blood electrolytes, pCO_2_, pO_2_, glucose, pH, standard base excess, and p-lactate (ABL Radiometer Medical, Denmark). Temperature, transcutaneous oxygen saturation, and MABP were measured at baseline and every hour after initiation of LPS infusion.

### HRV analysis

ECG data was recorded continuously during baseline and the 4 h after initiation of LPS infusion. ECG data was computer logged at 300 Hz (Datex Ohmeda S/5 collect, Finland).^32^ The raw ECG data from each animal was uploaded to Kubios Premium Software® (version 3.4.3), which automatically identified each R-wave. The ECG was divided into five-minute successive and non-overlapping epochs.^32^ Automatic beat correction was applied, and erroneous R-waves were corrected manually.^33^ Automatic beat correction was tolerated with a maximum of 3%. Epochs with cardiac arrhythmia and artefacts due to handling of the animal or ECG electrode replacement was excluded.^32^ HRV was analyzed in time-domain measures, non-linear measures, and frequency domain measures. The time-domain measures reflect the overall variability in the epoch and higher values are associated with low disease risk.^34^ Non-linear measures are based on geometric analyses and represent short-term and long-term variability within the epoch. In the frequency domain measures the variability is separated into components within pre-defined frequency ranges. Time domain measures analyzed included the standard deviation of normal-to-normal (meaning abnormal beats removed) intervals (SDNN) (ms), mean heart rate (HR) (beats/minute), and root mean square of successive differences in normal-to-normal heartbeats (RMSSD) (ms). Non-linear measures were analyzed using poincaré plots. This graphically presents successive RR-intervals where the line of identity represent points with identical successive RR-intervals.^19,33^ The standard deviation perpendicular to the line of identity (SD1) and along the line of identity (SD2) were included.^19,33^ Frequency domain measures were calculated using Fast Fourier transformation and estimated in pre-defined frequency bands as absolute power using the Welch’s periodogram.^19,33^ The following frequency measures were included; very low frequency (VLF), low frequency (LF), and high frequency (HF). There is no agreement on standardized frequency bands for neonates, but previous studies suggest the following bands which were applied in this study; VLF: 0.02–0.04 Hz, LF: 0.04–0.2 Hz, and HF: 0.2–2.0 Hz.^35,36^

### Statistical analysis

This study is a secondary analysis of previous experiments, which is in line with the *Reduction* principle in the 3 R’s statement of animal research.^37^ Therefore, no a priori power calculation was made. Temperature, MABP, systolic blood pressure, diastolic blood pressure, oxygen saturation measures and blood gas values were compared to baseline using one-way repeated measures ANOVA. Multiple comparisons were conducted by Fisher’s LSD test (no corrections for multiple testing). Complete blood cell count was compared between baseline and 4 h after initiation of LPS infusion using a paired *t*-test. HRV measurements from successive five-minute epochs were averaged to hourly means for each animal at baseline, 1 h, 2 h, 3 h, and 4 h after initiation of LPS infusion. All HRV measurements were log transformed to ensure normal distribution and a mixed-effects regression model was used to estimate crude relative changes in HRV at every hour after initiation of LPS infusion compared to baseline. A multivariable mixed-effects regression model including HR was used to investigate if the changes in HRV were due to changes in HR. A two-sided *p* < 0.05 was defined as statistically significant. Statistical analyses were made using GraphPad Prism (version 9.1.1 for MacOS) and Stata/SE 17.0.

## Results

**Fig. 2:**
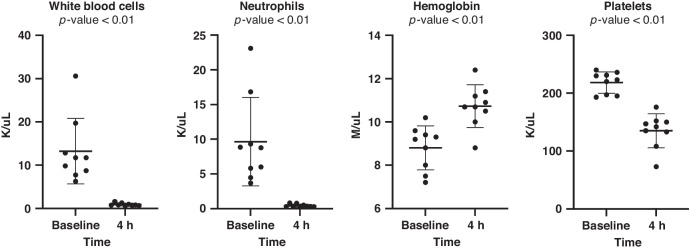
Blood cell counts at baseline and 4 h after initiation of LPS infusion. Baseline is compared to 4 h after initiation of LPS-infusion using paired *t*-test. Data are presented with superimposed mean and standard deviation. LPS Lipopolysaccharide from *Escherichia Coli*.

No animals were excluded prior to the experiments. ECG data was available from nine animals: six females and three males. The animals had a bodyweight between 1.6 and 2.1 kg. Systolic blood pressure was decreased at 3 h and 4 h and diastolic blood pressure was lower at 1 h, 2 h, and 4 h compared to baseline (Table 1). Blood gas analysis at 2 h and 4 h after initiation of LPS infusion showed small reductions in pH, pO_2_, base-excess, and glucose while p-lactate increased (Table 1). The administration of LPS was associated with lower white blood cell counts including neutrophile cell counts. Platelet count was also lower 4 h after initiation of LPS infusion, while hemoglobin concentration increased at 4 h compared to baseline (Fig. 2).

**Table 1:** Vital measures, arterial blood-gas values, and number of included epochs during the experiment.

	Baseline	LPS infusion	1 h	2 h	3 h	4 h	*p*-value
Vital values							
MABP (mmHg)	52 (7.2)		50 (7.0)	48 (4.3)*	48 (4.1)*	46 (6.3)*	<0.01
Systolic BP (mmHg)	68 (10.1)		70 (9.8)	65 (6.3)	62 (6.6)*	60 (9.0)*	<0.01
Diastolic BP (mmHg)	41 (5.6)		36 (5.2)*	37 (3.6)*	39 (4.5)	37 (4.7)*	0.02
SatO_2_ (%)	96 (1)		95 (2)	97 (1)	96 (2)	96 (2)	0.78
Temperature (°C)	38.6 (0.8)		38.9 (0.5)	39 (0.2)	38.8 (0.2)	38.8 (0.2)	0.50
Arterial blood gas							
pH	7.5 (0.1)			7.4 (0.0)*		7.4 (0.0) *	<0.01
pCO_2_ (kPa)	4.7 (0.5)			5.1 (0.4)		5.6 (0.3)*	<0.01
pO_2_ (kPa)	13.5 (2.2)			10.9 (0.7)*		10.2 (1.8)*	<0.01
Lactate (mmol/L)	0.8 (0.1)			1.3 (0.3)*		1.2 (0.3)*	<0.01
Base excess (mmol/L)	3.9 (2.7)			1.3 (2.4)*		0.9 (3)*	<0.01
Glucose (mmol/L)	5.7 (0.6)			4.3 (0.8)*		5.0 (0.5)*	<0.01
Number of epochs							
Number of epochs	11 (9-12)		12 (11-12)	12 (11-12)	12 (11-12)	11 (4-12)	0.12

Parametric data are presented as mean values with standard deviation. Non-parametric data are presented as median with inter-quartile range. Mean values of baseline are compared to every hour after LPS infusion using repeated measures one-way ANOVA presented with a corresponding *p*-value.

*LPS* Lipopolysaccharide from *Escherichia Coli*. *BP* Blood pressure.

*Indicates statistically significant difference (<0.05) when comparing baseline to the individual hour.

### HRV

**Fig. 3:**
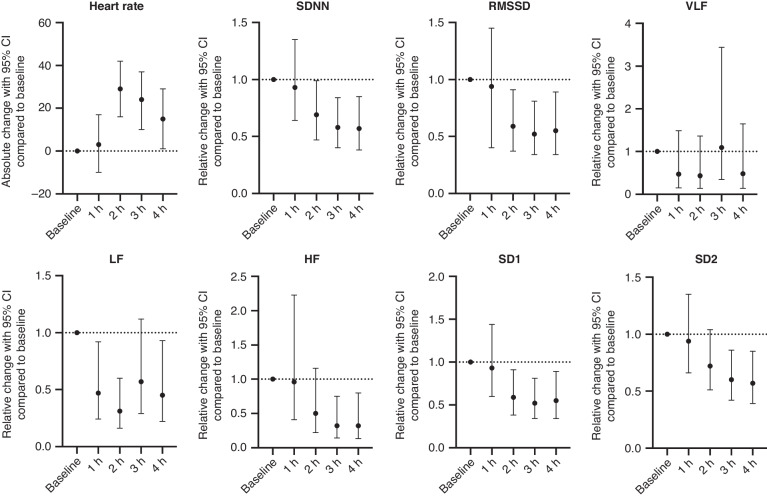
Relative changes in HRV. HR is presented as the absolute change with 95% CI for every hour after initiation of LPS infusion compared to baseline. HRV measures are presented as relative change with 95% CI for every hour after initiation of LPS infusion compared to baseline. The change from baseline to every hour is analyzed using a mixed effects regression model. LPS Lipopolysaccharide from *Escherichia Coli*. HR Heart rate in beats/minute, SDNN the standard deviation of normal-to-normal intervals, RMSDD the root mean square of successive differences in normal heartbeats, VLF power in very low frequency, LF power in low frequency, HF power in high frequencies, SD1 standard deviation perpendicular to the line of identity, SD2 standard deviation along the line of identity.

No differences were found in the number of included epochs between the different hours (Table 1). Reasons for exclusions of epochs are presented in Table 2. HR increased significantly 2 h, 3 h, and 4 h after initiation of LPS infusion with peak at 2 h. SDNN, RMSSD and SD1 showed an inverse pattern with relative reductions at 2 h, 3 h, and 4 h after initiation of LPS infusion compared to baseline. Lowest relative changes were at 2 h, 3 h and 4 h for RMSSD, SD1 and SDNN, respectively (Fig. 3). VLF did not change during LPS infusion. HF and SD2 were both lower at 3 h and 4 h after initiation of LPS infusion compared to baseline. LF already decreased 1 h after initiation of LPS infusion, then increased to a similar level as baseline at 3 h, and then decreased again at 4 h (Fig. 3). Data is also presented in table format in Supplementary Table 1 and as raw data in Supplementary Fig. 1.

**Table 2:** Overview of excluded epochs.

Reason for exclusion	Total number	% of excluded epochs	% of all epochs
Cardiac arrhythmia	13	19%	3%
Artefact due to handling of the piglet	23	34%	5%
Automatic beat correction over 3%	32	47%	7%
Total excluded epochs	68		14%

Total number of included epochs *n* = 472.

**Fig. 4:**
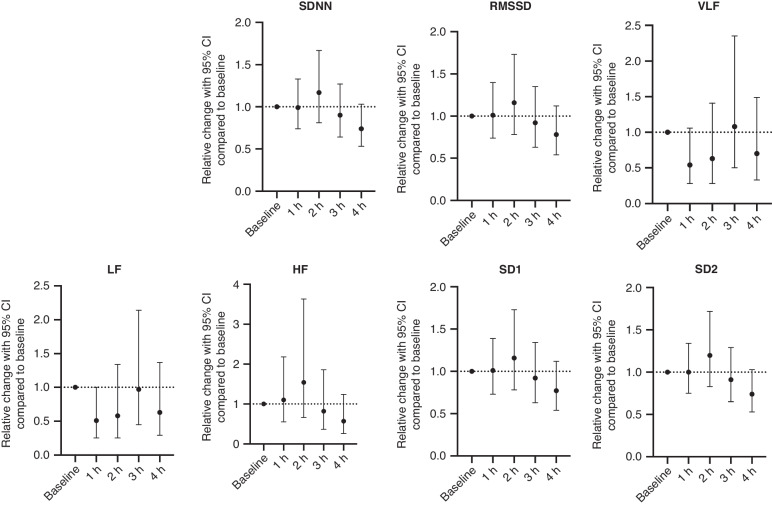
HR included in a multivariable mixed effects regression model comparing each hour after start of LPS infusion to baseline. HR is included in the model to investigate if the changes in the HRV measures are explained by changes in HR. HRV measures are presented as relative change with 95%CI for every hour after initiation of LPS infusion compared to baseline. The change from baseline to every hour is analyzed using a multivariable mixed effects regression model including HR. LPS Lipopolysaccharide from *Escherichia Coli*. HR Heart rate, SDNN the standard deviation of normal-to-normal intervals, RMSDD the root mean square of successive differences in normal heartbeats, VLF power in very low frequency, LF power in low frequency, HF power in high frequencies, SD1 standard deviation perpendicular to the line of identity, SD2 standard deviation along the line of identity.

When adding HR to the mixed effects regression model, no changes in any HRV variable over time (Fig. 4) was observed. Data is also presented in table format in Supplementary Table 2.

## Discussion

In a term newborn piglet model on sepsis established by continuous infusion of LPS, we show increased HR and decreased HRV as early as 2 h after initiation of LPS-infusion. The findings of reduced HRV suggest that HRV could be of value in early diagnosis of sepsis in term newborns. However, the systemic inflammation also caused increased HR which explained the reductions in HRV in the multivariable model.

Systemic inflammation during infection is thought to be modulated by neural reflex circuits.^38^ Infection is sensed and then efferent cholinergic nerves modulates the immune response through e.g., cell membrane receptor expressions and cytokine production.^38^ Both HR and HRV is partially controlled by cholinergic activity of the vagal nerve.^39,40^ Previous studies have described a complex association between HR and HRV as inversely correlated depending on the balance in the autonomic regulation.^20,41,42^ We showed the reduced HRV after induction of systemic inflammation was explained by the increase in HR, suggesting HR to be equally relevant as a potential biomarker of neonatal sepsis as the HRV measures. The lack of a clear definition of what constitute abnormal HR among neonates limits the clinical value of HR as a biomarker. Tachycardia in neonates may be due to several factors including pain, fever, hyperthermia, and other medical conditions in addition to sepsis. However, these limitations in using HR as a biomarker of neonatal sepsis also apply to HRV. No normal value of HRV has been agreed upon and inter- and intra-individual variations may be large, especially within the first days of life.^43,44^ We found large inter-individual variations at all time points impeding using HRV to predict a sepsis time point. For both HR and HRV, values should therefore not only be interpreted in relation to reference values but as the individual course of HR and HRV values starting with individual baseline values.^44^ However, obtaining reliable baseline values may be difficult in early onset sepsis. Furthermore, HRV may be affected by temperature, pain, other medical conditions, and drugs.^20,45^ Thus, neither HR nor HRV can stand alone in the detection of neonatal sepsis.^23,46,47^

Changes in HRV characteristics has been associated with increased risk of death and in neonates with VLBW. HRV monitoring was associated with reduced mortality from 10% to 8%.^22,48^ Maturation of the autonomic nervous system differs by gestational age and results from the preterm and VLBW population cannot be extrapolated directly to term newborns.^49^ In populations of both term and preterm newborns, tachycardia alone has been associated with increased risk of infection and bacteriemia.^50–52^ In a term ovine model, systemic inflammation induced by LPS was also associated with increased HR and reductions in LF, SD1, and SD2. SDNN, RMSSD, and HF were also reduced in LPS exposed animals, but findings were not statistically significant as in our study.^53^

The autonomous nervus systems maturation also changes with postnatal age.^44^ Therefore, the response in HR and HRV to early onset sepsis could differ from that in late onset sepsis. As the piglets in our study were born at term and were only 12 h old, our model is closer to term early onset sepsis. In one clinical study of term neonates, early onset sepsis was associated with high HR, while no association was found with HRV measures.^54^

In the neonatal intensive care unit (NICU) vital signs, such as HR, have been used in early warning systems to identify deterioration of the neonate.^55^ HR is an easily accessible objective marker routinely used by many clinicians often continuously monitored during the first days after admission, but the cut-off value of an abnormal HR seems to differ between neonatal departments.^46^ Alarm fatigue is a frequent problem due to the high level of variation in vital signs.^56,57^ The reduced mortality seen in neonates with VLBW that were monitored with HRV could be due to increased awareness on the newborn and more frequent clinical examinations due to a novel alarming device.^22^ Implementing continuous HRV monitoring as a permanent monitoring tool for term newborns, would potentially add more alarm fatigue in the NICU without adding to an equally careful attention to the HR. Comparison of HR and HRV’s ability as a warning tool to guide clinicians in sepsis diagnostics in term neonates would be relevant prior to implementing another monitoring device for these patients at the NICU.

The response in HRV to LPS infusion was measured for 4 h. When adding HR to the regression model, we found a tendency towards lower values of HRV at 4 h compared to baseline. Whereas HR peaked at 2 h and decreased closer to baseline at 4 h. It is possible that with an observation time longer than 4 h, HRV could decrease to lower values compared to baseline independent of HR. Meaning that, with longer duration of sepsis HRV may add to HR as a biomarker for sepsis. However, a late biomarker for sepsis is of less clinical relevance.

In the presented experiments, a sepsis-like condition was induced by infusion of LPS from E. coli. The endotoxemia causes a systemic inflammatory response comparable with that found in sepsis but different from the response triggered by a primary infectious agent. The experimental setting in animal research allows for standardized conditions, but the clinical immunopathology of sepsis in humans is likely more complex which could limit the possibility of extrapolating our findings to the clinical situation in neonates.^27^ Our findings of hypotension, decreased white blood cell count, including neutrophile count, decreased platelets, as well as an increase in hemoglobin concentration can be considered a proof of concept; i.e., that LPS infusion created a physiologic and biochemical systemic inflammatory response.^30^ Furthermore, a continuous infusion of LPS was administered throughout the experiment to limit the possible fluctuation in the endotoxin provided.

We administered LPS from a gram-negative bacterium. Thus, our findings only apply to gram-negative sepsis. Infection rates and mortality varies between geographical regions.^1,58^ The most common infectious agent in early onset sepsis in term neonates is GBS, which is a gram-positive bacterium; while E. coli may be the most common bacteria in preterm neonates in high resource settings.^3,13,59^ It could therefore be relevant to examine the effect of sepsis from gram-positive bacteria like GBS on HR and HRV.

The animals were anesthetized with propofol and fentanyl throughout the experiment. Previous studies have described changes in the autonomic tone during induction of general anesthesia with conflicting findings related to HR and HRV.^60,61^ Effects of anesthesia on HRV cannot be excluded, however, all animals were exposed to the same level of anesthesia at both baseline and during LPS-infusion.

Previous studies on late onset sepsis and HR characteristics in preterm newborns also investigated other HRV measures such as sample asymmetry and sample entropy. These HRV measures were not investigated in the present study. Therefore, we are unable to conclude on all HRV measures. Nguyen and colleagues found no associations between sample entropy and early onset sepsis in term newborns.^54^

## Conclusion

During a sepsis-like condition induced by continuous LPS infusion, HRV decreased after 2 h, 3 h, and 4 h. HR showed an inverse pattern with higher levels at 2 h, 3 h, and 4 h. All changes in HRV after inducing a systemic inflammation could be explained by HR. Reduced HRV may be an early biomarker of neonatal sepsis. However, an increase in HR alone could be an already available, more accessible, and interpretable biomarker of sepsis in term neonates.

## Supplementary information

Supplementary material

## Data Availability

Datasets generated in the presented study are available from the corresponding author on request.
